# Correction to: SARS-CoV-2 PCR-positive and PCR-negative cases of pneumonia admitted to the hospital during the peak of COVID-19 pandemic: analysis of in-hospital and posthospital mortality

**DOI:** 10.1186/s12879-021-06239-9

**Published:** 2021-07-19

**Authors:** Abduzhappar Gaipov, Arnur Gusmanov, Anara Abbay, Yesbolat Sakko, Alpamys Issanov, Kainar Kadyrzhanuly, Zhanar Yermakhanova, Lazzat Aliyeva, Ardak Kashkynbayev, Iklas Moldaliyev, Byron Crape, Antonio Sarria-Santamera

**Affiliations:** 1 Department of Medicine, Nazarbayev University School of Medicine, Kerey and Zhanibek Khans Street 5/1, Room 345, Nur-Sultan city, Kazakhstan; 2 Department of Emergency Medicine, Akhmet Yassawi University Medical Faculty, Turkestan, Kazakhstan; 3 Department of expertise, Social Health Insurance Fund branch of the Turkestan Region, Turkestan, Kazakhstan; 4 Department of Mathematics, Nazarbayev University School of Sciences and Humanities, Nur-Sultan, Kazakhstan; 5 Department of Preventive Medicine, Akhmet Yassawi University Medical Faculty, Turkestan, Kazakhstan

**Correction to: BMC Infectious Diseases 21, 458 (2021).**

**https://doi.org/10.1186/s12879-021-06154-z**

After publication of the original article [1], an error was identified in Fig. 3 b and c.

**Fig. 3:**
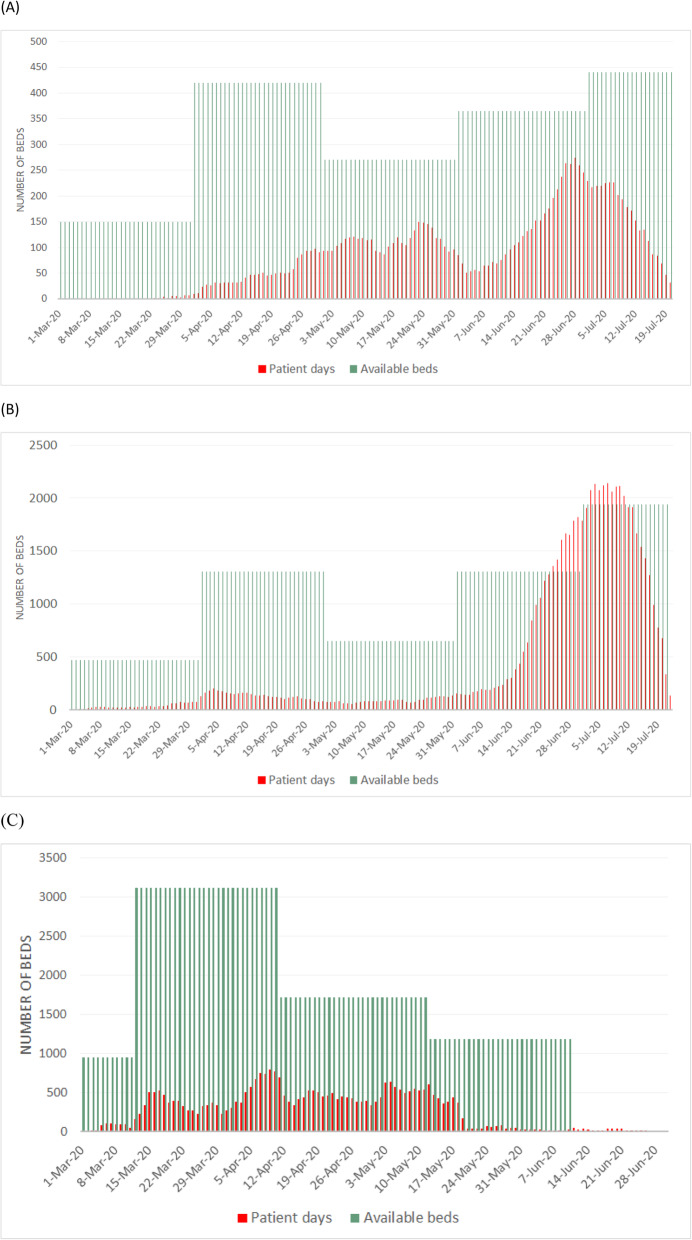
Dynamic change on hospital beds and admitted patients in infectious disease hospitals (**A**), provisional hospital (**B**) and quarantine hospitals (**C**)

The correct Fig. 3 is given below:

The original article has been corrected.
